# Oncologic outcome, side effects and comorbidity of high-intensity focused ultrasound (HIFU) for localized prostate cancer. A review

**DOI:** 10.1016/j.amsu.2020.05.029

**Published:** 2020-05-30

**Authors:** Francesco Ziglioli, Marco Baciarello, Giada Maspero, Valentina Bellini, Tommaso Bocchialini, Domenico Cavalieri, Elena Giovanna Bignami, Umberto Maestroni

**Affiliations:** a Department of Urology, University-Hospital of Parma, Via Gramsci, 14, Parma, Italy; b Anesthesia, Intensive Care and Pain Therapy Service, University-Hospital of Parma, Via Gramsci, 14, Parma, Italy

**Keywords:** Prostate cancer, Comorbidity, Side effects, High-intensity focused ultraosund, HIFU, Thermal ablation, Minimally-invasive procedure

## Abstract

**Introduction:**

Prostate cancer is considered one of the most important health problems. Due to the increased number of diagnosed patients and the inability to distinguish aggressive tumors, minimally-invasive procedures have become increasingly interesting. High-intensity focused ultrasound (HIFU) is an alternative option to radical surgery to treat prostate cancer. To date, however, data on side effects and comorbidities of this technique are still not conclusive.

**Methods and results:**

We reviewed the literature to concentrate on side effects and comorbidities of HIFU treatment of prostate cancer with the following key words: hifu, high intensity focused ultrasound, ultrasonic therapy, transrectal hifu, prostate ablation, side effects, comorbidities. MedLine and Embase via Ovid database were searched. Selection criteria were: English language, articles published between 2001 and 2015, case series including at least 100 participants and reported data on side effects and comorbidities. Sixteen uncontrolled studies were identified. No randomized controlled trials (RCT) were found in the literature comparing side effects and comorbidities of HIFU to other routine approaches to prostate cancer treatment.

**Conclusion:**

HIFU seems to be a promising minimally-invasive treatment for low- and intermediate-risk prostate cancer, especially for patients who are unfit for radical surgery. Prospective studies with longer follow-up periods and RCT are required to properly assess the impact of side effects and comobidities related to the HIFU technique in comparison with other therapies to treat prostate cancer.

## Highlights

• HIFU is a promising minimally-invasive treatment for prostate cancer, especially in patients with low- and intermediate-risk disease.
• To date, the most proper indication to HIFU is for patients who are not fit for, or are unwilling to undergo, radical surgery.
• The most common complications are impotence, urinary incontinence, acute urinary retention and urethral fistula.
• High-intensity focused ultrasound is a safe and effective procedure.

## 1 Introduction

The advent of PSA testing more than two decades ago has improved early detection of prostate cancer, leading to more men being diagnosed and treated.

Interestingly, it is still controversial whether the increased detection and treatment of prostate cancer has led to increased overall survival rates. Data from two long-term screening studies were published in the last few years and reported conflicting results. The Prostate, Lung, Colorectal and Ovarian screening concluded that there is no difference between men who were screened and men who were not screened [1]. On the other hand, the European Randomized Study of Screening for Prostate Cancer found a 20% reduction in the mortality rate in screened men [2].

Moreover, we cannot distinguish between tumors that will progress and lead to mortality and tumors that will not cause complications and are clinically insignificant. For this reason, there has been recent interest in organ-sparing therapies able to control local cancer with low invasiveness and morbidity and low impact on the quality of life.

Over the last years, minimally invasive procedures have emerged as management techniques in-between the surgical approach (Radical Prostatectomy) and watchful waiting. Different energy types and different methods of application have been developed to achieve the trifecta outcome (oncologic efficiency, continence and potency) [3], such as radiofrequency, cryotherapy, brachytherapy and high-intensity focused ultrasound (HIFU).

The aim of this review is to describe the principles of HIFU and to provide an overview of recent data on side effects and comorbidities related to the HIFU technique.

## 2 Materials and methods

### 2.1 Principles of HIFU

Lynn et al. proposed the focused ultrasound technique in 1942 [4,5], but it was firmly established in the 1950s, thanks to the work by Frank and William Fry, and initially used for ablating brain tissue [6,7]. One of the first investigators who conducted trials on this technique applied to human beings was S. Madersbacher [8].

The crucial impetus for the HIFU technique was the development of modern radiological imaging, such as diagnostic ultrasound (US) or magnetic resonance imaging (MRI), which allow non-invasive therapy guidance.

To date, only HIFU treatments of prostate cancer, uterine fibroids and, to some extent, the palliative ablation of bone metastases have found clinical acceptance, while in other pathologies, such as tumors of breast, kidney or liver, the numbers of treated patients remain small.

HIFU uses high-power, highly-focused ultrasound beams that are targeted to converge on a specific point within the body. This technique is also referred to as ultrasonic ablation, sonablation or focal ultrasound surgery. The ultrasound beam causes vibration, thus creating heat [9]. An analogy has been made with focusing the sun's rays through a magnifying glass to start a fire [10].

The source of HIFU is a spherical piezoelectric transducer able to produce ultrasonic energy focused on a fixed point. The transducer has the property of changing its thickness in response to an applied voltage, thus creating an acoustic ultrasound wave with a frequency equal to that of the voltage applied. Frequencies used for HIFU therapy cover a 3–4 MHz range. Depending on the ultrasound frequency, site-intensity ranges between 1300 and 2200 W/cm^3^ [[11], [12], [13]].

The thermal effect relies on the absorption of ultrasound energy by the tissue and its conversion into heat. A temperature of 75 °C can be achieved with 1s treatment, well above the level to denature protein (41 °C–43 °C) and sufficient for coagulative necrosis [14].

The lesions produced by the HIFU technique are elliptical with a volume between 50 and 300 mm^3^. They have also been defined as “cigar-shaped” [15].

By combining single lesions, larger target volumes can be ablated without gaps. Between single shots, a pause time is needed in order to prevent tissue boiling and bubble formation, which might distort the US-targeted area.

Focused ultrasound allows a well-circumscribed lesion to be obtained in the focal point without damaging the intervening tissues. The tissue layers outside the ablated area remain unaffected. Since the sharpness of such induced tissue necrosis is comparable to a surgeon's sharp incision, the therapy has also been termed Focused Ultrasound Surgery (FUS) [16]. Therefore, this technique provides the advantage of a transrectal treatment with prostate destruction, minimizing the risk of rectal injury [17].

By increasing the intensity of the waves and focusing them on a single point, HIFU allows the deposition of a large amount of energy into the targeted tissue, resulting in its destruction through cellular disruption and coagulative necrosis [18].

Two mechanisms of tissue damage are involved: thermal effect and cavitation [19].

The thermal effect is due to the conversion of ultrasound energy into heat. Tissue damage due to the thermal effect can be classified into three groups: hyperthermia that can destroy malignant cells with low temperatures (41–49 °C) during an extended period (>10 min); coagulation, consisting in necrosis of tumor tissue; and vaporization inducing tissue necrosis and charring (temperature >100 °C) [20].

Cavitation is the result of the interaction of ultrasound and water microbubbles. This interaction leads to microbubbles vibration and their dissolution within prostate tissue. When the bubbles reach the size of resonance, they suddenly collapse and produce high-pressure shock waves, thus destroying adjacent tissue [21,22]. The dynamics of cavitation bubble clouds generated at the tissue boundary in continuous HIFU fields has been experimentally investigated by high-speed photography [23].

Two HIFU devices are currently available, the Ablatherm (EDAP TMS SA, Vaulx-en-Velin, France) and the Sonablate device (Focus Surgery Inc, Indianapolis, IN, USA), which have been in use since 1993 and 1995, respectively. The differences between Ablatherm and Sonablate mainly concern patient positioning, treatment algorithm, imaging and technical details.

### 2.2 The ablation procedure

HIFU is performed through a computerized surgical device equipped with a treatment table, an ultrasound treatment system connected to an endorectal probe, a safety infrared ray detector, a refrigeration system keeping rectal mucosa below 14 °C and a monitor to set and control the treatment procedure through echographic screening. The single piezoelectric crystal alternates between high-energy power for ablation and low-energy for ultrasound imaging [24].

The treatment is performed under spinal anaesthesia. The procedure can be personalized in order to obtain ideal treatment settings: ultrasound frequency, shot duration and waiting time between shots may be modified.

HIFU-induced lesions are visible using standard ultrasound as hyperechoic areas. To date, MRI is considered the gold standard for HIFU efficacy assessment as gadolinium enhanced.

T1-weighted images can clearly show the necrosis extent [25].

### 2.3 Literature search and selection

**Fig. 1:**
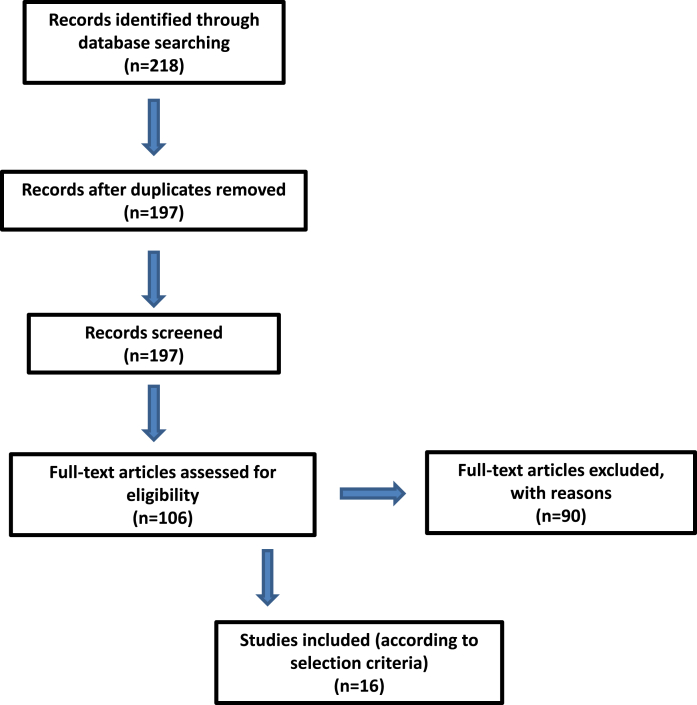
Literature search and selection.

We reviewed the literature focusing on side effects and morbidity of HIFU treatment for prostate cancer with the following key words: hifu, high intensity focused ultrasound, ultrasonic therapy, transrectal hifu, prostate ablation, side effects, comorbidity. MedLine and Embase via Ovid database were searched. Selection criteria were: English language, articles published between 2001 and 2015, case series including more than 100 participants and reporting data on oncologic outcome, side effects and morbidity related to the HIFU treatment. All studies that did not meet the inclusion criteria were excluded. Literature search was conducted from 25th to June 28, 2019. Literature search and selection is summarized in Fig. 1.

The metholodogy of the review is in accordance with the PRISMA criteria [26] and the quality of the review was self-evaluated using the AMSTAR-2 criteria [27]. The overall confidence rate in the results of the review is moderate according with the AMSTAR-2 criteria.

The study was registered with the Research Registry. The unique identifying number (UIN) of the study is 5325.

## 3 Results

We identified 16 case series assessing HIFU as a primary or salvage therapy option in prostate cancer [[28], [29], [30], [31], [32], [33], [34], [35], [36], [37], [38], [39], [40], [41], [42], [43]]. Results on side effects and comorbidity are shown in Table 1. The number of patients included in the case series ranged from 100 to 1002, giving a total of 5094 patients, with a mean age of 65.3 years, ranging from 64.1 to 72.7 years; it was not possible to determine how many patients underwent redo-HIFU. Also, some reports seemed to refer to the same group of patients, with different follow-up duration. Whenever possible, double citations were eliminated. Most patients underwent one treatment.

**Table 1:** Side effects and comorbidity of HIFU.

	n	ED	Incontinence G1	Incontinence G2	Incontinence G3	Incontinence	Fistula	Urethral stenosis	AUR	UTI	More than 10d CA	Post-op Pain
Blana et al., 2004 [28]	146	57.2	n/r	n/r	n/r	8 (5,8%)	0	17 (11,7%)	n/r	6 (4,1%)	4 (2,7%)	1 (1,4%)
Thuroff et al., 2003 [29]	402	13	43 (10,6%)	10 (2,5%)	6 (1,5%)	59 (14,6%)	5 (1,2%)	14 (3,6%)	35 (8,6%)	55 (13,8%)	0	n/r
Gelet et al., 2001 [30]	102	61 (59,8%)	9 (8,8%)	10 (9,8%)	4 (3,9%)	23 (22,5%)	1	17 (17%)	5 (5%)	n/r	9 (9,1%)	2 (2%)
Chaussy & Thuroff 2003 [31]	271	84 (31%)	17 (6,2%)	8 (3,1)	5 (1,7%)	30 (11%)	n/r	40 (14,7%)	n/r	66 (24,3%)	40 (14,8%)	n/r
Poissoinier et al., 2007 [32]	227	39 (17,2%)	9 (3,9%)	3 (1,3%)	1 (0%)	13 (5,7%)	0	12 (5,3%)	9 (3,9%)	2 (0,8%)	0	3 (1,3%)
Ahmed HU et al., 2009 [33]	172	n/r	13 (7,6%)	n/r	1 (0,6%)	14 (8,2%)	0	52 (30,2%)	n/r	41 (23,8%)	n/r	n/r
Mearini L et al., 2009 [34]	163	n/r	18 (16%)	18 (16%)	1 (0%)	(52) 32%	1 (0%)	24 (15%)	n/r	n/r	n/r	n/r
Murat FJ et al., 2009 [35]	167	n/r	n/r	54 (32,5%)		54 (32,5%)	5	33 (20%)	13 (7,8%)	6 (3,5%)	n/r	n/r
Blana et al., 2008 [36]	163	34 (44,7)	10 (6,1%)	3 (1,8%)	0	13 (7,9%)	0	40 (24,5%)	n/r	13 (7,8%)	n/r	3.7
Uchida T et al., 2009 [37]	517	33 (28,9)	5 (0,8%)	0	0	5 (0,8%)	6 (0,9%)	105 (20,3%)	84 (16,2)	n/r	n/r	n/r
Maestroni et al., 2012 [38]	100	90 (90%)	7 (7%)	4 (4%)	0	11 (11%)	1 (1%)	0	4 (4%)	n/r	0	n/r
Pfeiffer D et al., 2012 [39]	191	n/r	51 (26,5%)	12 (6,3%)	2 (1,6%)	65 (34,4%)	3 (1,6%)	n/r	n/r	51 (26,5%)	n/r	n/r
Ganzer R et al., 2013 [40]	538	126/202 (62,3%)	93 (17,3%)	15 (2,8%)	3 (0,7%)	111 (33%)	4 (0,7%)	n/r	152 (28,3%)	55 (10,2%)	n/r	n/r
Thuroff S & Chaussy C 2013 [41]	704	316 (45%)	n/r	n/r	n/r	28 (4%)	0.23%	0	4.6%	2.1%	n/r	0.27%
Berge V et al., 2013 [42]	130	50.3	n/r	n/r	n/r	13 (9,9%)	n/r	18 (13,8%)	n/r	14 (10,8%)	7.6	n/r
Crouzet S et al., 2013 [43]	1002	577 (57,7%)	187 (18,7%)	50 (5%)		237 (23,7%)	4 (0,4%)	90 (9%)	166 (16,6%)	39 (3,9%)	n/r	n/r

Erectile function after treatment was reported in 11 studies. One study, where it was not possible to distinguish patients treated with HIFU from patients treated with other minimally invasive techniques, like cryotherapy, brachytherapy and vascular-targeted photodynamic therapy (VTP) was excluded.

However, erectile function impairment ranged from 13% to 90%.

Urinary incontinence ranged from 4% to 34.4% and was reported from all studies. In the majority of studies, the group of patients who reported urinary incontinence was subdivided in grade I (range 0.8%–26.5%), grade II (range 1.8%–16%) and grade III (range 0%–3.9%). In 3 studies only overall urinary incontinence was reported. In two studies grade II and grade III incontinence were reported together. In one study only grade I and grade III urinary incontinence was reported and grade II incontinence was not mentioned.

Mean catheter duration was ≥10 days in four of 16 studies, ranging from 12.7 to 24.8 days. Data on urinary tract infections (UTIs) were reported in 12 studies. None of them reported complications of UTIs. UTIs consequent to HIFU ranged from 0.8% to 26.5%.

Acute urinary retention (AUR) was not systematically considered as a HIFU-related complication by many Authors, however it ranged from 3.9% to 28.3%.

The only major complication related to the procedure was recto-urethral fistula. The rate of this complication ranged from 3.6% to 30.2%. Two Authors reported the absence of this complication in their series. Two of 16 Authors did not report any data on this complication. The management of this complication was conservative treatment (prolonged catetherization) or open reconstructive surgery. Urethral stenosis was also reported, ranging from 0 to 30.2%. The Authors, however, did not provide details on the severity of the stenosis in the studies considered. Also, it was not possible to investigate the relation between urethral stenosis and acute urinary retention. Another complication of HIFU is the occurrence of Urinary Tract Infection (UTI), in the post-operative time. UTIs ranged form 0.8%–24.3% and data on UTIs were reported by 12 of 16 studies. Post-operative pain was reported by 5 on 16 studies, with no mention of the scale used to assess pain severity.

Oncologic data are reported in Table 2. Gleason score ranged from 2 to 10, the vast majority of patients being ≤7. In most series, the D'Amico risk classification was used, with a prevalence of low.risk group.

**Table 2:** 

Author	n	Age	Mean follow-up (months)	PSA (ng/mL)	PSA nadir (ng/mL)	Failure criterion	nADT (n)	TUR-P pre HIFU (n)	DFSR
Blana et al., 2004 [28]	146	66.9	22.5	7.6	0.07	Positive biopsy or PSA ≥0,2 ng/mL	63 (43%)	n/r	54 (3y)
Thuroff et al., 2003 [29]	402	69.3	58.1	10.9	1.8	ASTRO	n/r	n/r	n/r
Gelet et al., 2001 [30]	102	70.8	76	8.38	0.57	Phoenix	8 (7,8%)	n/r	66 (5y)
Chaussy & Thuroff 2003 [31]	271	n/r	n/r	n/r	n/r	n/r	n/r	175 (64,6%)	n/r
Poissoinier et al., 2007 [32]	227	68.8	27	6.99	n/r	biopsy, PSA >1 ng/mL	76 (33,4%)	175 (77%)	66 (5y)
Ahmed HU et al., 2009 [33]	172	64.1	11.5	8.3	n/r	PSA ≤0,5 ng/mL	50 (29%)	None	n/r
Mearini L et al., 2009 [34]	163	72	23.5	7.3	0.15	Phoenix, biopsy	None	None	n/r
Murat FJ et al., 2009 [35]	167	68	18	6.9	n/r	Phoenix ASTRO	95 (56,9%)	n/r	53 (3y)
Blana et al., 2008 [36]	163	72	57.6	7.3	11	Phoenix ASTRO	None	n/r	78,1 (5y)
Uchida T et al., 2009 [37]	517	68	24	9.2	n/r	Phoenix ASTRO	343 (66,3%)	n/r	72 (5y)
Maestroni et al., 2012 [38]	100	72.7	24	18.2	0.12	Phoenix	17 (17%)	100 (100%)	78 (3y)
Pfeiffer D et al., 2012 [39]	191	69.7	52.8	7.2	0.09	Stuttgart	81 ((4,2%)	92 + 2 (4,2%)*	62,8 (5y)
Ganzer R et al., 2013 [40]	538	67.7	n/r	11.2	0.4	Phoenix ASTRO	196 (36,4%)	416 (77,3%)	61 (10y)
Thuroff S & Chaussy C 2013 [41]	704	68.4	n/r	9.9	1.7	Phoenix ASTRO	61 (4,2%)	528 (75%)	99 (10y)
Berge V et al., 2013 [43]	229	65.9	27	7.9	n/r	n/r	n/r	n/r	n/r
	1002	71	76.8	7.7	0.14	Phoenix ASTRO	392 (39,1%)	939 (93,7%)	97 (10y)

*92 patients underwent TUR-P and 2 patients underwent adenomectomy.

PSA before treatment ranged from 6.9 ng/mL to 18.2 ng/mL. Mean pre-treatment PSA was 8.51 ng/mL (SD).

Between 0% and 66.3% received neoadjuvant androgen deprivation therapy (nADT) and between 0% and 100.% underwent TUR-P (Trans-Urethral Resection – Prostate) before or in combination with HIFU. In some series, data about pre-HIFU TUR-P were not reported. In one case [37], 2 patients underwent adenomectomy before HIFU.

The vast majority of the case series used Phoenix criteria to define failure and to assess the oncological outcome for the treatment. On one case, Stuttgart criterion was used [37]. In one case, we found that the criterion used to define oncological failure consisted in finding two PSA ≥0.5 ng/mL [31] and, in another case, PSA ≥ 1 ng/mL [30].

Mean follow-up time was 32.7 months, ranging from 11.5 to 76.8 months.

PSA nadir was reported in most studies and ranged from 0.07 to 1.8 ng/mL.

Desease-free survival rate (DFSR) was reported in 11 out of the 16 identified series, while it was not well defined or not reported at all in 5 series. When patient stratification in risk groups was reported, the highest DFSR was found in the low-risk group.

Prostate biopsies were taken at 3 or 6 months after HIFU in the vast majority of cases.

## 4 Discussion

Depending on tumor stage and life expectancy, the European Association of Urology (EAU) and the American Association of Urology (AUA) recommend radical prostatectomy, external beam radiation therapy (EBRT) and active surveillance as standard treatment options for patients with localized prostate cancer [44,45].

HIFU has emerged as an alternative therapeutic option in patients with clinically localized prostate cancer, who are not suitable for Radical Prostatectomy [46]. Although the medical associations of France, United Kingdom and Italy approve HIFU as primary and salvage treatment for prostate cancer, the AUA and the EAU do not recommend its routine use [[47], [48], [49]]. This is due to the overall lack of data about long term follow-up and HIFU comparison to conventional therapy options.

However, because biopsy strategies and imaging techniques can detect a higher number of tumors, there is growing interest in minimally invasive therapies, especially for patients who are unsuitable for major surgical procedures.

Despite the fact that HIFU technique has been used for many years, data reported in the literature are still controversial and evidence of its routine use is not available. Moreover, there are no randomized controlled trials comparing the HIFU technique to radical prostatectomy or other minimally invasive techniques for the treatment of prostate cancer.

Life expectancy for men has increased at least 4 years in the last 24 years, while the age of prostate cancer detection has decreased on average 10 years, thus leading to diagnosis at an earlier stage of the disease. This change in age and extent of disease at diagnosis has revealed limitation in conventional curative modalities of treatment, such as the risk of aggressive cancer recurrence and the risk of long term morbidity and its impact on the Quality of Life (QoL), as it is argued by Chaussy CG et al. [50]. For this reason, minimally invasive techniques for the treatment of prostate cancer, like HIFU, look to be an attractive option. In this view, a full knowledge of the oncologic outcome as well as the long term side effects of this treatment should be considered of paramount importance.

Our literature search identified 16 valuable studies, but there is no common agreement about the methodology to measure the effectiveness, side effects and comorbidity related to HIFU treatment.

Actually, there is not a common criterion to define failure. In the most majority of studies, failure was assessed according to the ASTRO criteria [51], generally used for defining failure after radiotherapy. Even if the Stuttgart definition, a PSA increase of 1.2 ng/mL above the PSA nadir value [52], has been validated specifically for HIFU, it is used to assess failure in a minority of studies.

When ASTRO and Stuttgart criteria were not used, the effectiveness of HIFU treatment was assessed using surrogate outcome, like negative prostate biopsy or biochemical-free survival rate. However, it remains questionable whether surrogate outcomes correlate with patient-relevant outcomes [53].

The majority of data on comorbidity and side effects of HIFU are reported in studies whose primary endpoint was the efficacy of the technique and its oncologic outcome.

In addition, Authors used different criteria to assess side effects and in some cases data collection was not standardized by using questionnaires or objective parameters.

Erectile Dysfunction (ED) is reported by all Authors as a consequence of the treatment. When the literature for ED after HIFU is reviewed, it ranges from 13% to 90%. The most important determinant of post-operative erectile function status proved to be pre-operative erectile function [54].

The most common side effect of HIFU treatment is voiding dysfunction and urinary retention caused by edema, necrosis and consequent bladder outlet obstruction. Trans-Urethral Resection of the Prostate (TUR-P) is likely to reduce the impact of these side effects, thus improving the quality of life in the post-operative time [31]. Data reported by Chaussy CG and Thuroff S on patients who underwent TUR-P before HIFU treatment and patient who did not undergo any disobstructive procedure before treatment conclude that TUR-P reduces the postoperative catheter duration, the risk of Urinary Tract Infections (UTIs), the risk of strictures and stenosis and the risk of grade I and grade II urinary incontinence [31]. Unfortunately, these study is on a small number of patients and results cannot be generalized.

Last but not least, major complications are also reported by the majority of series. The most common – even if rare - major complication was recto-urethral fistula. Not all Authors reported how this complication was managed, but from the data available we can state that in some cases it required open surgery, when not amenable of conservative treatment.

Another point is redo-HIFU. Unfortunately, it is not possible to determine the number of patients who underwent retreatment. The reasons for repeating treatment were technical problems, large prostate and residual tumor or recurrence. Although the number of repeated HIFU certainly demonstrates the safety of the procedure, it also generates confusion when data of different studies are compared and side effects of the HIFU technique are evaluated.

In addition, if the technique efficacy seems encouraging in terms of disease-free survival rate and in terms of number of failures in the 5y, 7y and 10y follow-up periods, it is still difficult to determine the burden of side effects in the long term. Going through the literature, we found a study proposing an index to preview the risk of recurrence (PSA rising) from variables available before treatment [55], but unfortunately there is not any tool to preview the burden of comorbidities, which would open the way to a discussion among investigators about patients selection and indications to HIFU treatment.

## 5 Conclusions

High-intensity focused ultrasound is considered a promising minimally-invasive treatment for prostate cancer, especially in patients with low- and intermediate-risk disease. To date, the most proper indication to HIFU is for patients who are not fit for, or are unwilling to undergo, radical surgery.

The most common complications are impotence, urinary incontinence and acute urinary retention. As a major complication, urethral fistula is the most reported.

## Provenance and peer review

Not commissioned, externally peer reviewed.

## Ethical approval

Not required.

## Sources of funding

None.

## Consent

Not applicable.

## Author contribution

Francesco Ziglioli. Study concept or design. Writing the paper.

Marco Baciarello Study concept or design. Writing the paper.

Giada Maspero Writing the paper.

Tommaso Bocchialini Data analysis and interpretation.

Domenico Cavalieri Data analysis and interpretation.

Valentina Bellini. Data analysis and interpretation.

Umberto Maestroni Study concept or design. Supervision.

## Registration of research studies

Name of the registry: Research Registry.

Unique Identifying number or registration ID: 5325.

Hyperlink to the registration (must be publicly accessible): https://www.researchregistry.com/browse-the-registry#home/

## Guarantor

Francesco Ziglioli and Umberto Maestroni.

## Declaration of competing interest

None.
